# Multi-defect risk assessment in high-speed rail subgrade infrastructure in China

**DOI:** 10.1038/s41598-024-56234-8

**Published:** 2024-03-06

**Authors:** Jinchen Wang, Yinsheng Zhang, Luqi Wang, Yifan Sun, Jingyu Zhang, Jianlin Li, Sen Li

**Affiliations:** 1 Key Laboratory of Geological Hazards On Three Gorges Reservoir Area of Ministry of Education, China Three Gorges University, Yichang, 443002 Hubei People’s Republic of China; 2 School of Environmental Science and Engineering, Huazhong University of Science and Technology, Wuhan, 430074 Hubei People’s Republic of China; 3 School of Artificial Intelligence and Automation, Huazhong University of Science and Technology, Wuhan, 430074 Hubei People’s Republic of China; 4 College of Hydraulic and Environmental Engineering, China Three Gorges University, Yichang, 443002 Hubei People’s Republic of China

**Keywords:** High-speed rail, Subgrade defects, Environmental factors, Risk assessment, Machine learning, Civil engineering, Environmental impact

## Abstract

This study addresses the escalating risk of high-speed railway (HSR) infrastructure in China, amplified by climate warming, increased rainfall, frequent extreme weather, and geohazard events. Leveraging a georeferenced dataset of recent HSR defects obtained through an extensive literature review, we employ machine learning techniques for a quantitative multi-defect risk assessment. Climatic, geomorphological, geohydrological, and anthropogenic variables influencing HSR subgrade safety are identified and ranked. Climatic factors significantly impact frost damage and mud pumping, while geomorphological variables exhibit greater influence on settlement and uplift deformation defects. Notably, frost damage is prevalent in the northeast and northwest, mud pumping along the southeast coast, and settlement and uplift deformation in the northwest and central areas. The generated comprehensive risk map underscores high-risk zones, particularly the Menyuan Hui Autonomous and Minle County sections of the Lanzhou-Urumqi HSR, emphasizing the need for focused attention and preventive actions to mitigate potential losses and ensure operational continuity.

## Introduction

High-speed railways (HSR) provide efficient and convenient services for passenger transportation, playing an important role in stimulating the regional economic and social development^1,2^. As of 2020, the length of HSR tracks in China has reached 38,000 km, spanning various terrains and climatic zones^3,4^. Consequently, constructing railways becomes inevitable in environmentally challenging areas. The subgrade of HSR constitutes a soil structure directly exposed to the natural environment, and the impact of climate change on the long-term performance of HSR subgrades cannot be ignored. According to the “The Global Climate In 2015–2019” released by the World Meteorological Organization and the “2019 China Climate Bulletin” released by the China Climate Center, with global warming, extreme rainfall events are increasingly frequent in China. Under the action of high-frequency train loads, the subgrade is more prone to various defects such as settlement^5^, uplift deformation^6,7^, frost damage^8^, and mud pumping^9^. The occurrence of these defects is uncertain and poses a significant threat to human life and property. Therefore, investigating their spatial pattern, defect mechanisms, and vulnerability indicators is of great significance for the early warning of defects and the planning of new HSR lines.

Traditional risk assessment methods for HSR subgrades primarily utilized qualitative methods included the Analytic Hierarchy Process (AHP) and expert scoring method, while quantitative models such as the Information Value Model and Frequency Ratio Model relied heavily on extensive data for support. Machine learning has gained increasing attention because of its advantages such as robust generalization ability, high processing efficiency, and the ability to handle large datasets. For example, Wang et al. (2022) utilized two deep learning (DL) algorithms, convolutional neural network (CNN) and deep neural network (DNN), to map landslide susceptibility on a branch line of the Sichuan-Tibet Railway^10^. Liu et al. (2018) quantitatively analyzed the susceptibility of existing and planned railroad systems in China to rainfall-triggered multi-hazards using Random Forest (RF) and historical disaster events from 1980 to 1998^11^. Huang et al. (2022) integrated four machine learning models, i.e., Bayesian Networks (BN), Decision Tables (DTable), Radial Basis Function Networks (RBFN), and Stochastic Gradient Descent (SGD), to delineate landslide-prone zones in order to reduce the risk of the construction, maintenance, and transportation of the railroad in Sichuan^12^. Huang et al. (2023) organized seismic damage data of bridges, then used RF to predict seismic damage levels, and used a two-parameter normal distribution function to draw empirical susceptibility curves for seismic damage risk assessment^13^. Sresakoolchai et al. (2023) developed a novel intelligent automated system based on machine learning pattern recognition for detecting and predicting the deterioration of railroad turnouts exposed to flood conditions^14^. Although machine learning gained frequent application in railroad safety risk assessment, it tends to focus on the disturbance of railroad operational status by external small-scale disasters, and overlooked the impact of structural changes in the subgrade itself on overall railroad risk in the climatic and geographical environment.

Selecting driving factors of defects is a crucial step in predicting the risk of railway subgrade failure in China under long-term environmental changes. Previous studies indicate that subgrade defects are influenced by rainfall, temperature, geological conditions, and land use patterns. For example, rainfall induces the absorption of water by soft, weak mudstone, resulting in arching on the subgrade. Water infiltration from the surface into the subgrade soil reduces its shear strength, leading to various defects in the subgrade. Thus, the occurrence of defects is the outcome of multiple factors in specific conditions. Zhang et al. (2016) conducted freeze–thaw tests on soil–cement mixtures from a construction site to explore the freeze–thaw susceptibility of closed and open systems^15^. The findings revealed that the frost heave rate was influenced by the initial water content before freezing and the replenishment of moisture during the freezing process. Wan et al. (2022) established a vehicle-track-subgrade vertical dynamic coupled analysis model was established using ABAQUS^16^. The study found that debonding easily occurred between the end of the base plate and the surface of the subgrade. As rainwater continuously infiltrated and saturated the surface of the subgrade, fine particles gradually migrated upward under the action of train loads and accumulated on the surface of the subgrade, leading to mud pumping. Although previous studies have explained the processes underpinning defect occurrence through field experiments and numerical simulations, the extent to which driving factors affect defects remains unknown. Further investigation is needed to clarify the correlation between different environments and different defects.

This study utilized a novel dataset of historical subgrade defect occurrences to reveal their relationship with environmental factors for large-scale infrastructure risk assessment for HSR in China, employing machine learning methods. The main objectives of this study are to (i) investigate the diverse impacts of environmental factors on multiple common subgrade defects, and (ii) spatially predict the co-occurrence risk of subgrade defects. Spatial risk maps of common road defects were constructed, offering a decision-making basis for the safe management and spatial planning of HSR in China.

## Methods and data

### Historical HSR subgrade defect occurrences in China

**Figure 1:**
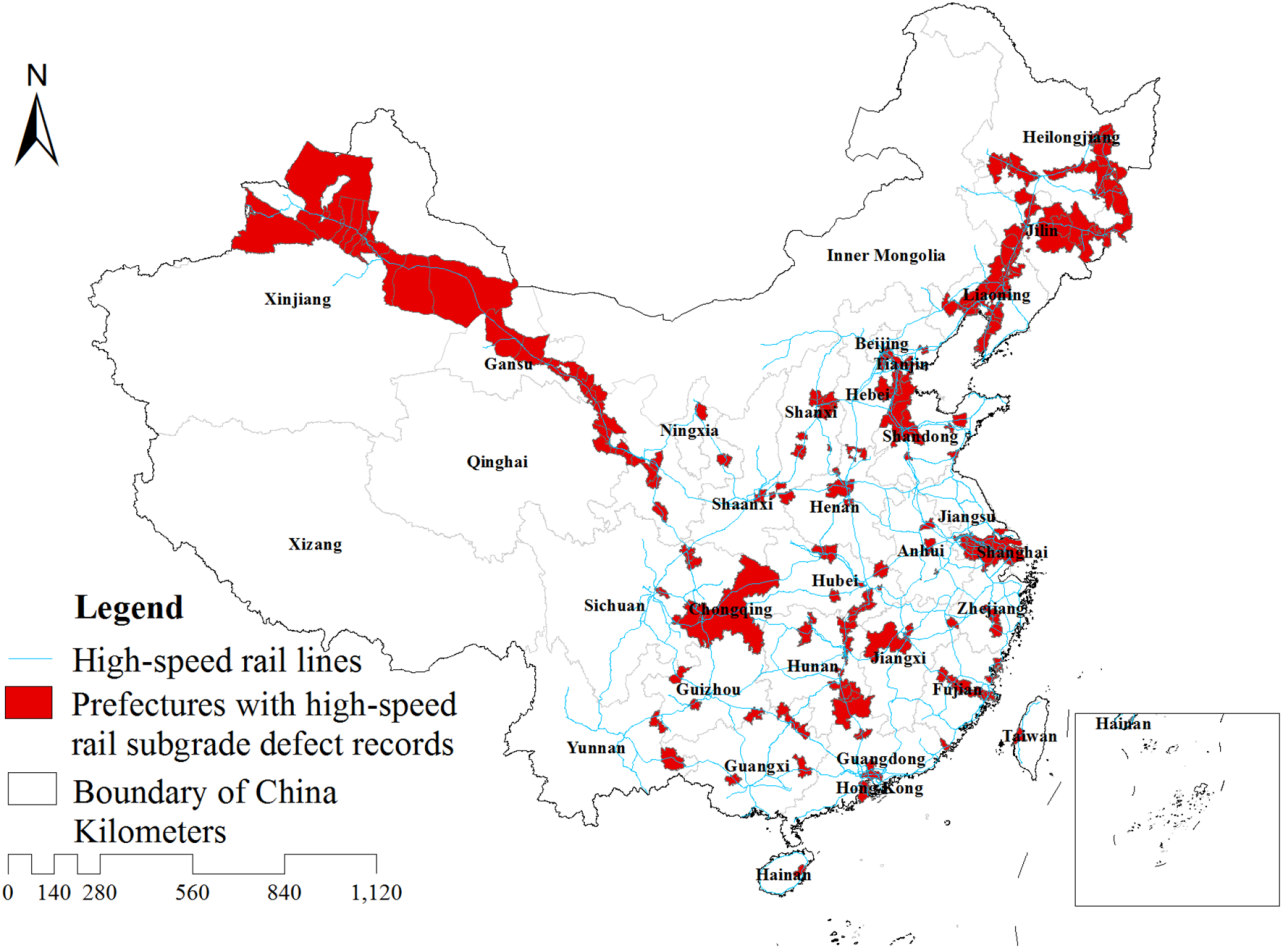
Distribution of HSR subgrade defect records in China.

We recently compiled an extensive georeferenced dataset of historical HSR subgrade^17^. The dataset was sourced from 24,735 peer-reviewed literature published from 1999 to 2022 in both Chinese and English, and a quality control procedure was applied to remove duplicates and ensure accuracy^18,19^. Subsequently, a total of 661 georeferenced event records of eight defect types were selected, crossing provincial, municipal, county, township, and smaller scales. Notably, subgrade settlement (settlement values ranging from 5 to 2300 mm), frost damage (frost heave values ranging from 4 to 50 mm), uplift deformation (ranging from 5 to 122 mm), and mud pumping exhibit the longest reporting history among the identified disease types. These definitions are detailed in Table 1. The distribution of HSR subgrade defect records across Chinese prefectural-level administrative regions is illustrated in Fig. 1.

**Table 1:** Definition of major types of HSR subgrade defect.

Type of defect	Definition
Subgrade settlement	Settlement refers to the vertical deformation that occurs over a small or extensive area due to inadequate compaction of subgrade soil, insufficient depth of foundation treatment, damage between piles, creep of underlying soil layers, or regional settling
Frost damage	In cold regions, subgrade and its protective structures experience uneven frost heave under low temperature conditions, leading to issues like tilting and cracking of protective structures
Mud pumping	This defect occurs in areas with poor drainage. Repeated vibrations from train traffic cause softening or thixotropic liquefaction of the sub-ballast, leading to the formation of mud slurry
Uplift deformation	This happens when expansive soils or rocks within the subgrade or its base react with external moisture, causing the subgrade to arch upwards

The results indicate that the occurrence of these defects can be closely related to local climate and geological environment. For example, frost damage events are concentrated in the temperate zone of China, which is characterized by long and cold winters and high humidity throughout the year. The presence of pore water in the soil particles in the subgrade freezes and forms ice layers, resulting in soil displacement and subgrade frost heave. Mud pumping events are concentrated in the southeastern part of China, where frequent heavy rainfall occurs, causing a large amount of rainwater to infiltrate into the subbase and reduce its bearing stiffness. Under the high-frequency dynamic loads of trains, mud pumping and, in severe cases, subgrade settlement can occur. Subgrade swelling and upheaval are closely related to the slight expansion of the fill material used. Within the same climatic zone, multiple diseases often coexist, making the subgrade condition more complex.

### Environmental driving factors

#### Climate variables

*Average annual rainfall*: Rainfall may alter the engineering properties of subgrade materials, thereby influencing the stability of the subgrade^20^.

*Consecutive 5-day rainfall*: This data serves as an index reflecting extreme rainfall^21^.

*Number of days with maximum temperature exceeding 35 degrees celsius*: This data can serve as an indicator reflecting extreme high temperatures^16^.

*Annual freezing days*: Annual freezing days quantify the number of days in a region where water freezes, and it is a key factor influencing the occurrence of frost damage on roadbeds^15^.

*Wind speed*: Strong winds may erode road shoulders, leading to a reduction in subgrade width, with sleepers/track panels exposed, thereby affecting the stability of the railway track^22^.

#### Geomorphological variables

*Elevation*: Elevation defines the highest and lowest points within a region and is reported to relate to the occurrence of various defects, such as, a number of defects have been reported on the Menyuan-Minle section of the Lanzhou-Urumqi HSR at high altitude^15^.

*Slope and aspect*: HSR subgrades may have varying slopes, resulting in different temperatures inside and outside the subgrade, potentially leading to uneven settlement^23,24^. The slope gradient may have an impact on the flow of moisture, thereby disrupting the drainage of the subgrade^25^.

#### Geohydrological variables

*Rock hardness*: Harder rocks can provide better support for HSR subgrade^3^.

*Distance to fault*: Geological faults provide pathways for groundwater and surface precipitation, which can affect subgrade^26^.

*Soil texture*: Subgrade defects can be associated with the types and properties of surrounding soil^27^.

*Average distance to river*: The presence of rivers increases the amount of groundwater in the surrounding geological environment, thus affecting the performance of subgrade^28^.

*Average distance to lake*: Lakes increase the amount of groundwater in the surrounding geological environment, which can impact the performance of subgrade^8^.

#### Anthropogenic variables

*Land use*: Land use indirectly influences the occurrence of subgrade defects. Extracting groundwater in urban areas can lead to subgrade defects, while areas with multiple rock types can enhance the strength of subgrade and reduce settlement^29^.

*Average distance to road*: Road construction, as a human activity, can have an impact on railway lines^30,31^.

#### Variable sources and preparation

The average annual rainfall, consecutive 5-day rainfall, number of days with maximum temperature exceeding 35 degrees Celsius, annual freezing days, and wind speed data were sourced from the National Earth System Science Data Center (http://www.geodata.cn/), with a spatial resolution of 0.25° and a time range from 2007 to 2016. We obtained annual average rainfall data through kriging spatial interpolation. The remaining factors were summarized within specified regions using ArcGIS's zoning statistical function, displaying the data values in tabular form; Elevation data were obtained from the Geospatial Data Cloud (http://www.gscloud.cn) with a resolution of 30 m. Slope and aspect data at a 30 m resolution were derived using ArcGIS software's slope function and aspect analysis tool; Land use data were sourced from the Institute of Geographic Sciences and Natural Resources Research, Chinese Academy of Sciences (http://www.igsnrr.ac.cn), with an accuracy of 30 m. We calculated land use area within specified regions using ArcGIS’s zoning statistical function; The road, river, and lake data were extracted from OpenStreetMap. We calculated the average shortest distance from railway lines to these features using ArcGIS; Rock hardness and fault data were provided by the Geological Survey Cloud of the China Geological Survey Bureau (https://geocloud.cgs.gov.cn/). We categorized geological formations into different intervals to determine the average rock hardness within the region. The average shortest distance from railway lines to geological faults was calculated using ArcGIS. Soil texture data were sourced from the Harmonized World Soil Database (version1.2) (https://www.fao.org/home/en/), and we selected four soil attributes, including soil drainage capacity, soil composition, soil effective water storage capacity, and soil depth through filtering processes.

### Methods

#### Data processing

The variables were standardized using the StandardScaler module, and the hyperparameters of the RF were optimized using grid search to build a screening model^32^. To streamline and enhance model performance, the recursive feature elimination method was used to remove the environmental variables with minimal contribution^33,34^. Specifically, a RF model was iteratively established 18 times, eliminating the least important environmental factors in each screening process based on their contribution. A criterion was set to prevent the incorrect elimination of important factors, ensuring that the contribution of the eliminated factors did not exceed 0.005. The adjusted remaining predictor factors were reintroduced into the model. Finally, 55 factors, out of the initial 73, for each type of defect were retained to construct the risk prediction model. All the factors are shown in Table 2.

**Table 2:** Detailed description of factors.

Variable categories	Variable subclasses		Number of variables	Definitions and units
Climate variables	Rainfall		2	Average annual rainfall (mm)
				Consecutive 5-day rainfall (mm)
	Temperature		2	Number of days with maximum temperature exceeding 35 degrees Celsius(day)
				Annual freezing days(day)
	Wind speed		1	(m/s)
Geomorphological variables	Elevation		1	(m)
	Slope		1	(°)
	Aspect		1	(°)
Geohydrological variables	Rock hardness		1	(Pa)
	Distance to fault		1	(Km)
	Soil texture	Soil composition	13	Clay(heavy), silty clay, clay, silty clay loam, clay loam, silt, powdery sandy loam, sandy clay, loam, sandy clay loam, sandy loam , loamy sand, sand
		Effective water storage capacity	7	The United Nations Food and Agriculture Organization (FAO) evaluates the capacity of soil units to store water at a specific depth, dividing it into 7 levels: 150 mm/m, 125 mm/m, 100 mm/m, 75 mm/m, 50 mm/m, 15 mm/m, and 0 mm/m
		Soil depth	4	For all soil units, the reference depth is typically set at 100 cm. However, for Rendzinas and Rankers in the FAO-74 classification, and for Leptosols in the FAO-90 classification, the reference soil depth is set at 30 cm; for Lithosols in FAO-74 and Lithic Leptosols in FAO-90, the reference depth is set at 10 cm, and also at 0 cm
		Drainage class	7	Excessively drained, Well drained, Moderately well drained, Moderately drained, Somewhat poorly drained, Poorly drained, Very poorly drained
	Average distance to river		1	(Km)
	Average distance to lake		1	(Km)
Anthropogenic variables	Land use		29	Paddy fields, drylands, forested lands, shrublands, vegetable woodlands, other forested lands, high cover grassland, medium cover grassland, low cover grassland, rivers, canals, lakes, reservoirs, permanent glacial snowfields, mudflats, beachland, urban land, rural residential, industrial and commercial construction land, sandy land, Gobi, saline and alkaline land, marshland, bare land, bare rock, other unused land, alpine desert, tundra, marine
	Average distance to road		1	(Km)

#### Random forest modelling

The RF model is one of the most commonly used integrated algorithms in applied Machine Learning studies^35,36^. It utilizes repeated independent sampling to extract multiple samples from the original dataset and constructs decision trees for each sample. These decision trees are then aggregated and combined by voting, taking each decision tree as a member to achieve classification and prediction. In this study, the Random Forest algorithm emerges as a crucial tool in predicting the risk of subgrade defects in HSR infrastructure. Its capacity to process extensive datasets with various input variables and is robust against overfitting make it exceptionally suited for this task. Furthermore, as a non-parametric model, RF does not require assumptions about any specific form of relationship between variables, offering a significant advantage in examining the complex and not yet fully understood interplay between environmental factors and subgrade defects. Applying RF allows us to capture non-linear relationships and variable interactions that traditional statistical methods might overlook. Finally, RF is widely recognized and effective in identifying and determining variable importance. As a result, this approach has been successfully applied in the past for mapping landslides, debris flows, and many other types of disasters^28,29,37^.

RF calculates the decrease in Gini index ${D}_{Gk}$ by evaluating the evaluation factor *k* during node splitting. The importance of the evaluation factor *k* is determined by summing up ${D}_{Gk}$ of all nodes in the forest and taking the average over all trees. This measure represents the percentage of the average decrease in Gini index for the evaluation factor in relation to the total average decrease in Gini index for all factors. It is calculated according to Eq. (1):

1 $${P}_{K}=\frac{\sum_{h=1}^{n}\sum_{j=1}^{l}{D}_{Gkhj}}{\sum_{k=1}^{m}\sum_{h=1}^{n}\sum_{j=1}^{l}{D}_{Gkhj}}$$

where *m*, *n*, and *l* represent the total number of evaluation factors, the number of classification trees, and the number of nodes in a single tree, respectively. ${D}_{Gkhj}$ refers to the decrease in the Gini index of the *j*th node in the *h*th tree for the *k*th evaluation factor. ${P}_{K}$ denotes the importance level of the *k*th evaluation factor among all evaluation factors.

When constructing the RF models, the dataset was divided into a 7:3 ratio for training and validation. To enhance the robustness of model predictions and quantify the uncertainty, we employed an ensemble of 50 models trained on separate bootstraps of the dataset. The hyperparameters of each of the 50 individual models were determined using grid search, with random combinations of parameters, while all other tuning parameters were set to their default values. The combination with the highest average accuracy across the models was selected as the optimal parameter choice for the model. Furthermore, a five-fold cross-validation strategy was employed, whereby the training dataset was divided into 5 equal subsets, with 4 subsets used for model training and the remaining subset utilized for testing. This five-fold process was repeated iteratively, rotating the testing subset, in order to fully leverage all the training data for model training and testing while mitigating the impact of overfitting. To minimize the influence of randomness, each type of pathology was subjected to 50 models. Each one of these 50 models predicted the environmental risk on a continuous scale ranging from 0 to 1, and the final prediction graph was generated by calculating the average prediction across all models.

The model’s classification accuracy is analyzed using the Receiver Operating Characteristic (ROC) curve^38–40^, depicting the true positive rate on the vertical axis and the false positive rate on the horizontal axis. Greater accuracy in model classification is indicated by a higher true positive rate and a lower false positive rate. The ROC curve is generated by plotting the true positive rate (proportion of correctly identified defect samples) against the false positive rate (proportion of falsely identified non-defect samples).

#### Integrated risk map generation

The integrated HSR infrastructure risk assessment involves a holistic analysis that encompasses multiple subgrade defects that are most commonly reported in China, such as settlement, frost damage, uplift deformation, and mud pumping. This approach takes into account the cumulative impact of various factors—including climatic conditions, geomorphological features, geohydrological characteristics, and human activities—on the subgrade's safety. In regions where the integrated risk scores are relatively high, an enhanced need for coordination and management emerges to effectively mitigate potential risk.

To quantify this integrated risk, we utilized the Random Forest (RF) model to evaluate the probability of each defect type occurring, averaging the outcomes across 50 iterations. Natural breakpoints were then utilized to divide each defect into four risk levels: low, low-medium, medium–high, and high^28,41,42^. Portions with average probability values greater than 0.6 for each defect were selected and assigned a value of 1; otherwise, they were assigned 0. Spatial coupling of the four defects was performed to produce a comprehensive risk map of railway subgrade defects in China The low, medium, high, and very high risk areas in the graph have values of 0, 1, 2, and 3, respectively, representing the risk level of the area.). It is noteworthy that this map displays regions with high risks for all four defects (probability values greater than 0.6), thus necessitating extra attention in HSR operations and new HSR planning. All distribution maps in the figure were drawn by ArcGIS (v10.7, www.esri.com).

## Results

### Evaluation of model predictive power

**Figure 2:**
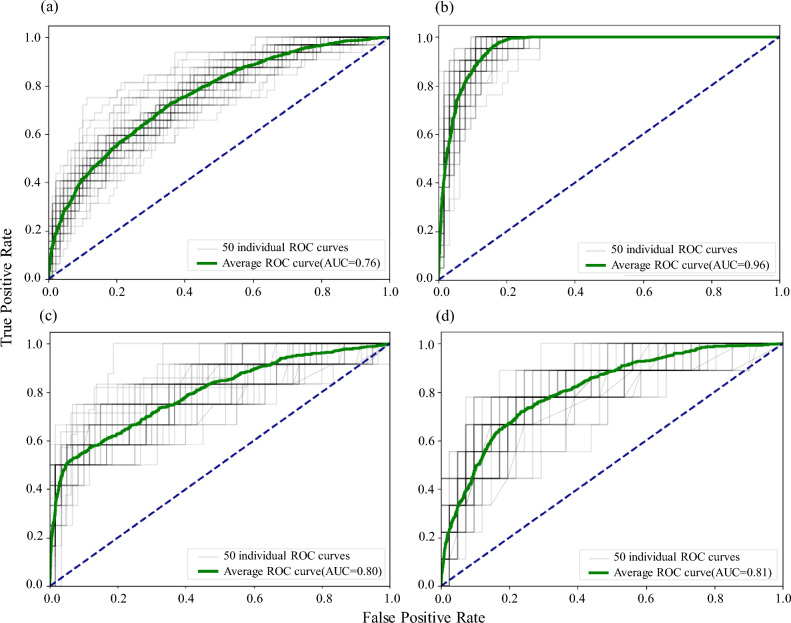
Model prediction evaluation using AUC values and ROC curve analysis: (**a**) subgrade settlement, (**b**) frost damage, (**c**) uplift deformation, and (**d**) mud pumping.

This study employed RF to evaluate the susceptibility of road defects in China, verified the training accuracy (success rate) using its ROC curve. The average AUCs for subgrade settlement, frost damage, uplift deformation, and mud pumping were obtained through 50 rounds of sampling, with values of 0.76, 0.96, 0.80, and 0.81, respectively, as shown in the Fig. 2. The green line represents the average ROC curve, while the black lines represent the 50 individual ROC curves. These results demonstrated that the RF model exhibited good prediction capabilities for generating risk maps of subgrade defects.

### Predicted high risk areas for different subgrade defect types

**Figure 3:**
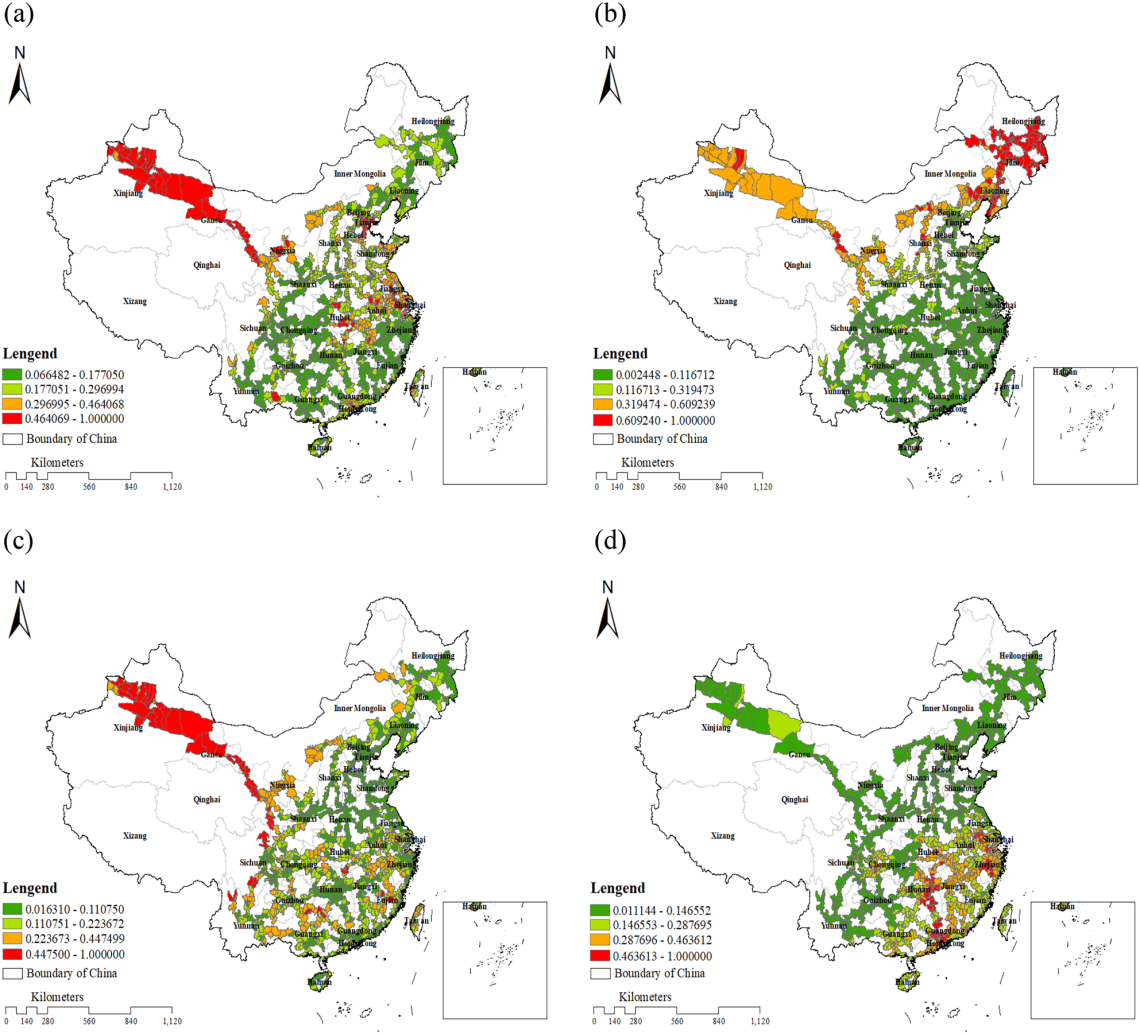
Predicted risk distribution of main HSR subgrade defects in China: (**a**) subgrade settlement, (**b**) frost damage, (**c**) uplift deformation, and (**d**) mud pumping. Mean are shown for each ensemble of 50 RF models.

Predicted high risk areas for settlement defects mainly concentrate on the Lanzhou-Urumqi HSR and the Shanghai-Nanjing Intercity Railway, with higher susceptibility in northwest and central China (Fig. 3a). Frost damage risks (Fig. 3b) were predicted to primarily concentrate on the Harbin-Dalian HSR, the Lanzhou-Urumqi HSR, with higher susceptibility in northeastern and western China. Areas prone to uplift deformation (Fig. 3c) were predicted to mainly concentrate on the Lanzhou-Urumqi HSR, and to mud pumping defects (Fig. 3d) primarily concentrate on the Shanghai-Nanjing Intercity Railway and the Wuhan-Guangzhou HSR, with higher susceptibility in southeast China.

**Figure 4:**
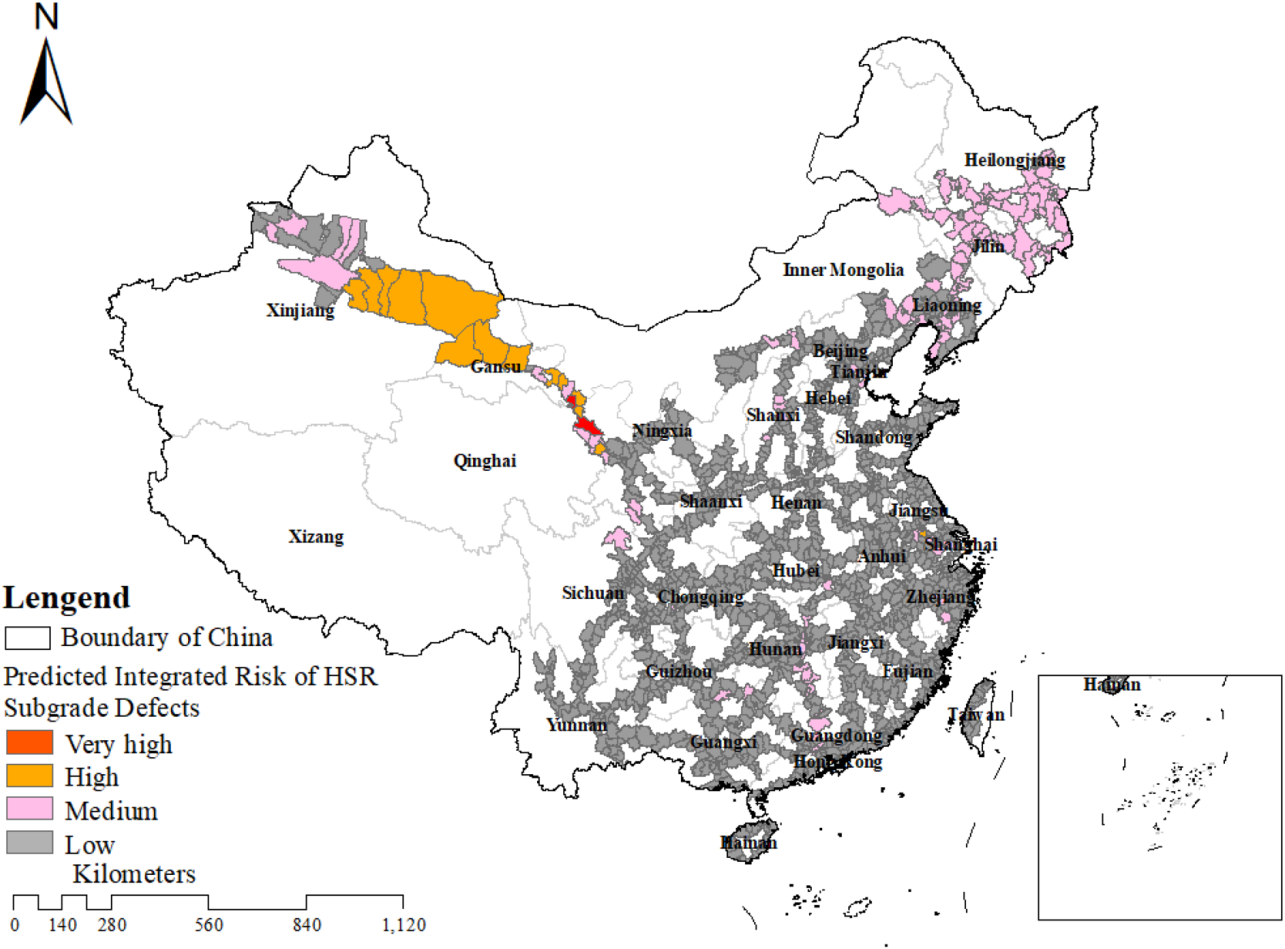
Integrated co-occurrence risk map of HSR subgrade defects in China.

In the integrated risk map for subgrade defects in China’s HSR (Fig. 4), the predominant occurrences of subgrade defects in China’s HSR are concentrated in the northeast, northwest, and central regions. The Lanzhou-Urumqi HSR has the highest likelihood of subgrade defects, which is closely correlated with the local climate and environmental conditions.

### Key environmental drivers of subgrade defect risk

**Figure 5:**
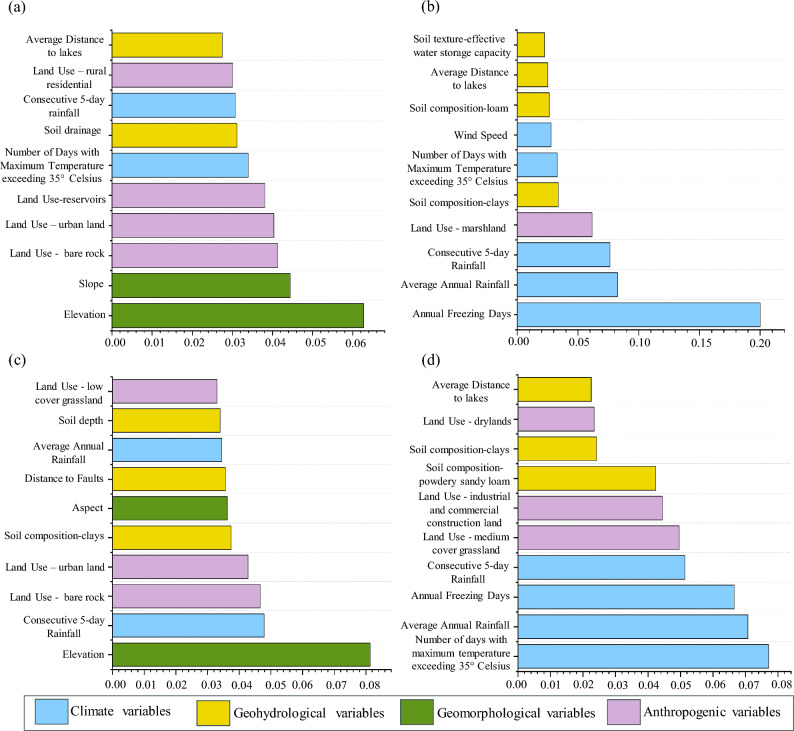
Importance map of defect factors of HSR subgrade foundations in China (**a**-**d**, subgrade settlement, Frost damage, uplift deformation, and mud pumping).

The occurrence of each defect is influenced by multiple influencing factors, each with varying degree of impact. Utilizing the “Gini coefficient” based on the RF model^43^, the average factor importance of 50 sets was calculated to generate the final factor importance ranking for each defect, as shown in the Fig. 5. We selected the top 10 most important factors for presentation. Regarding settlement defect, the importance factors included elevation, slope, and land use-bare rock, with importance values of 0.063, 0.044, and 0.041, respectively. For frost damage, the importance factors were number of freezing days per year, annual average rainfall, and continuous 5-day cumulative rainfall with importance values of 0.20, 0.082, and 0.076, respectively. For uplift deformation, the elevation, continuous 5-day cumulative rainfall, and land use-bare rock had importance values of 0.081, 0.048, and 0.047, respectively. For mud pumping, the driving factors were number of days with maximum temperature exceeding 35 degrees Celsius, annual average rainfall, and number of freezing days per year, with importance values of 0.077, 0.070, and 0.067, respectively.

## Discussion

### Climatic impacts

Our results indicates that meteorologically variables have a significant impact on subgrade defects, particularly in frost damage and mud pumping. The analysis prioritizes the identification of the most influential meteorologically variables associated with each defect.

Rainfall factors ranked among the top three in terms of importance, with the exception of settlement defects*.* The driving force behind the influence of rainfall on common subgrade defects lies in its capacity to increase the moisture content of the pavement soil. We have found that defects such as subgrade settlement, frost damage, mud pumping, and uplift deformation are intricately linked to the presence of water, which is consistent with the research results of many researchers^20,27,44–46^. In the case of frost damage, soil moisture undergoes crystallization into ice, filling soil voids during temperature drops, resulting in relative displacement of the subgrade particles. Mud pumping could be influenced by the softening of pore water pressure in the subgrade under train loads, making it highly susceptible to pumping and subgrade softening. Uplift deformation is associated with the expansion of expansive rock and soil in the subgrade expands upon water absorption. Related research has shown a significant correlation between the vertical uplift deformation rate of the pavement and the amount of atmospheric rainfall. Furthermore, extreme precipitation, indicated by the rainfall amount over 5 consecutive days, could exceed subgrade drainage capacity, elevating soil moisture and heightening susceptibility to defects.

We found that the annual freezing days have the greatest impact on the frost damage of the subgrade, which is consistent with the indoor experiments of subgrade permafrost and numerical simulations^25^, because the annual freezing days are closely related to the freeze–thaw cycle of the subgrade, which can lead to the occurrence of frost damage. The variable classified frozen soil in subgrade into three categories: instantaneous frozen soil, seasonal frozen soil, and permafrost. Permafrost, due to its long-term exposure to cold areas, is often in a state of freezing expansion. Seasonal frozen soil experiences thawing and settlement in summer and freezing expansion in winter. The recurring cycle of freezing expansion and settlement poses a significant risk of HSR subgrade defects. The damage to the subgrade in regions with repeated occurrences of such frozen soil is notably higher than in areas with permafrost. Instantaneous frozen soil generally experiences less freezing expansion. The effect on mud pumping mainly stems from the fact that after the freezing and thawing of the subgrade soil. The water content in the soil takes various forms, including ice crystals and residual moisture, which leads to a decrease in the drainage capacity of the subgrade and makes it difficult to drain moisture effectively. Consequently, poor drainage can result in mud pumping in the subgrade.

Extreme heat, represented by the total number of days with a maximum daily temperature exceeding 35 degrees Celsius, could impact the HSR infrastructure. High temperature can cause the rubber material at the interjoint of the ballastless track slab to harden and fatigue, resulting in the detachment of the interjoint interface and unevenness in the track. With repeated cycles of high-temperature and low-temperature alternation, the rubber material may even fracture, contributing to mud pumping defects.

### Geomorphological and geohydrological characteristics

Our results show that geomorphology had a significant impact on roadbed settlement and uplift deformation defects (Fig. 5), while the geohydrological factors showed comparatively less impact. This is inconsistent with the research findings of some researchers^11,47^, possibly because HSR has already avoided the risks caused by geohydrological variables during the design phase. On steeper slopes, especially during heavy rainfall, soil erosion is prone to occur on the subgrade surface. Soil erosion may accelerate the settlement process of the roadbed, affecting its stability. In areas with significant slopes, the speed of water flow may be higher, which could impact water infiltration and drainage. This may result in uneven distribution of moisture in the soil, thereby affecting the settlement behavior of the subgrade.

Moreover, with increasing altitude, temperature, precipitation, and atmospheric pressure may undergo significant changes, thereby affecting the stability of the subgrade. From a topographic perspective, high-altitude areas, characterized by steep slopes and complex terrains, yield significant variations in local climates between foothills and hinterlands. Therefore, a more detailed analysis of the region and a precise subgrade risk assessment based on the local climate are necessary.

The microscopic properties of clay minerals contribute to their capability to absorb water molecules on their surfaces. Frost damage to the subgrade only occurs when the water in the soil reaches or exceeds a certain threshold, making clay more likely to cause frost damage than other soils. As for the uplift deformation of the subgrade, when clay absorbs water, its volume will increase and expand, which may lead to up-arching of the roadbed.

### Anthropogenic influence

Our results show that anthropogenic variables have a significant impact on various subgrade defects, with the analyses emphasizing the most influential anthropogenic variables associated with most defects. Urban land use signifies the extent of anthropogenic interference, which includes the extraction of groundwater, the construction of underground facilities, mining and so on. When groundwater is extracted, the water pressure in the soil changes and the pumped water carries away fine particles from the soil, resulting in settlement of the subgrade. For example, in the Jakarta and Bandung areas along the Jakarta-Bandung High-Speed Rail, industrial activities and rapid population growth have resulted in the extensive extraction of groundwater, causing significant land subsidence that severely affects the operation of the high-speed train^48^. For the uplift deformation defect, in the high-density urban area, a large number of buildings. The area of bare rock reflects the surface area of land consisting of rocks. The bare rock areas may have good characteristics for water infiltration and drainage, which can slow down soil settlement through drainage. In addition, the scarcity of soil moisture may prevent the swelling of weak mudstone, thus reducing the occurrence of roadbed expansion defects and positively affecting the stability of the subgrade.

### Limitations and future improvements

Our study has several limitations that can be addressed in future research. Firstly, the defect data in this study are sourced from peer-reviewed literature, which ensures accuracy but may overlook some unreported defect data. Secondly, selecting the model’s hyperparameters poses significant challenges. The crucial parameters of the model are determined through trial and error using a network search method. If the search space is set inappropriately or potential solutions are overlooked, the optimal solution may not be found. Furthermore, while the Random Forest method is recognized for its strong predictive capability, it falls short in interpretability. Future research should consider employing specified analytical methods to further explore the casual relationships among various influencing factors and improve understanding of the mechanisms by which these factors impact high-speed rail infrastructure. Lastly, due to limited resources and capabilities, the selected influencing factors in this study may not be comprehensive. In future research, we can incorporate more reliable data, including media reports, government documents, and bidding information, to avoid overfitting caused by insufficient data. Additionally, we can explore methods for optimizing the model’s parameters and include HSR attribute factors to further enhance the model’s accuracy.

### Potential for application

This study applies a robust and effective machine-learning method for assessing the diverse defect risks inherent in China’s high-speed railway infrastructure. The practicality of the Random Forest method is not limited to specific geographic regions or infrastructure types; its powerful data processing capability and the ability to identify complex relationships between environmental factors grant it broad application potential. For instance, it can accommodate adjustments in environmental variables, such as rainfall and temperature variation, to suit various climatic zones (e.g., tropical, temperate, polar). Furthermore, this method can be applied to datasets for different infrastructures including roads, bridges, and tunnels, taking into account their unique risk factors and challenges. By fine-tuning the inputs to the algorithm, it is possible to precisely predict the specific risks faced by these different infrastructures, thereby providing a scientific basis for the design, construction, and maintenance of infrastructure.

### Policy recommendations

Global climate change, marked by temperature increases, intensified precipitation, and extreme events, threatens HSR safety and reliability, affecting infrastructure and surrounding environments^49–51^. Particularly vulnerable regions like Minle County and Menyuan Hui Autonomous County (Fig. 4), with seasonal frozen soils, face heightened subgrade defects due to disrupted thermal equilibrium. To address these concerns, several policy recommendations are proposed.

First, research and development efforts for HSR infrastructure should be intensified, focusing on enhancing resilience to climate change through developing materials and technologies that can withstand extreme weather conditions. Real-time monitoring and early warning of the seasonal frozen soil environment in the regions housing specific HSR projects, such as Lanzhou-Urumqi and Harbin-Dalian HSRs, should be strengthened. This aims to timely grasp the changes in the frozen soil environment and provide scientific basis for safe and stable operation of HSR projects. In the Far Eastern Railway in Russia, long-term monitoring of subgrade deformation, weather, and rock layers on railway sections located in permafrost areas has been implemented to mitigate the effects of extreme atmospheric precipitation^52^.

Moreover, the design standards of HSR projects should be revised to accommodate frozen soil climate conditions. Construction processes and methods should be optimized to ensure the safety and reliability of HSR projects in frozen soil areas during construction and operation. Emergency response plans and risk assessment systems for HSR must be established and enhanced in response to climate change. This includes augmenting early warning and response capabilities for extreme weather events, and effectively respond to the sudden risks brought about by climate change. Similarly, in Norway, a preparedness framework has been developed to assess and manage natural climate risks, aiming to reduce railway vulnerability and enhance resilience against the negative impacts of climate change. This includes emergency plans for trains include speed restrictions in high-risk areas and providing alternative transportation methods when tracks are obstructed^53^.

Lastly, strengthening safety promotion in areas along the HSR line should be emphasized. The government should fully utilize online methods such as government websites, television broadcasting, and new media, as well as offline methods such as home visits and setting up prominent warning signs, to proactively promote policies and regulations related to protecting the safety environment along the HSR line and reducing anthropogenic interference with HSR safety. In Sweden, particularly regarding the Varberg Railway, a study highlighted that human-induced groundwater extraction increases the risk of railway subsidence, suggesting the need for enhanced safety management measures along the railway lines^54^.

## Conclusions

This study quantitatively assesses the multi-subgrade defect risk in China’s HSR infrastructure, utilizing machine learning and historical defect occurrence data. Key environmental factors influencing subgrade defects, such as rainfall, freezing days, extreme temperature, land use, slope, and altitude, are identified, providing valuable insights for HSR planning. Furthermore, spatial analysis further reveals the distribution characteristics of different defects across various regions in China, particularly pointing out high-risk areas like the Menyuan Hui Autonomous and Minle County sections of the Lanzhou-Urumqi HSR, which require increased attention and preventative measures to minimize potential losses and ensure operational continuity.

For high-risk areas and types of defects, we recommend intensifying R&D efforts for HSR projects to develop materials and technologies capable of withstanding extreme weather conditions; optimizing design standards and construction methods for HSR projects, especially under permafrost climate conditions; establishing and improving emergency response plans and risk assessment systems for HSR to address sudden risks posed by climate change; and enhancing safety promotion along HSR lines to reduce human interference and ensure the safe and stable operation of HSR. While focused on China’s HSR, the methods are adaptable to railway infrastructure risk assessment globally, with challenges remaining in incorporating engineering design characteristics and evolving climate change impacts. Further research is needed to address these challenges.

## Data Availability

The data that support the findings of this study are available from the corresponding author upon reasonable request.

## References

[1] Bian X, Duan X, Li W, Jiang J (2021). Track settlement restoration of ballastless high-speed railway using polyurethane grouting: Full-scale model testing. Transp. Geotech..

[2] Qizhou H, Xin F, Lishuang B (2021). Natural disaster warning system for safe operation of a high-speed railway. Saf. Environ..

[3] Chen R-P (2014). Recent research on the track-subgrade of high-speed railways. J. Zhejiang Univ. Sci. A..

[4] Zhao J, Liu K, Wang M (2020). Exposure analysis of Chinese railways to multihazards based on datasets from 2000 to 2016. Geomat. Geomat. Nat. Haz. Risk..

[5] Xiao SG, Yan QR, Chen W (2012). Characteristics of settlement and assessment methods of engineered structures under certain high-speed railway tracks in china. Adv. Energy Mater..

[6] Xue Y, Wang Q, Ma L, Yu Y, Zhang R (2023). Mechanisms and controlling factors of heave in summer for high-speed railway cutting: a case study of Northwest China. Constr Build Mater..

[7] Dai Z (2021). Long-term uplift of high-speed railway subgrade caused by swelling effect of red-bed mudstone: case study in Southwest China. B Eng. Geol. Environ..

[8] Zhang T, Shen W-B, Wu W, Zhang B, Pan Y (2019). Recent surface deformation in the Tianjin area revealed by Sentinel-1A data. Remote Sens-Basel..

[9] Xu P, Sun Q-X, Liu R-K, Wang F-T (2013). Key equipment identification model for correcting milepost errors of track geometry data from track inspection cars. Transp. Res. C Emer..

[10] Wang, S. et al. Evaluation of landslide susceptibility of the Ya’an–Linzhi section of the Sichuan–Tibet Railway based on deep learning. Environ. Earth Sci. **81**, (2022).

[11] Liu K, Wang M, Cao Y, Zhu W, Yang G (2018). Susceptibility of existing and planned Chinese railway system subjected to rainfall-induced multi-hazards. Transp. Res. A Pol..

[12] Huang, J., Ling, S., Wu, X. & Deng, R. GIS-Based comparative study of the Bayesian network, decision table, radial basis function network and stochastic gradient descent for the spatial prediction of landslide susceptibility. Land. **11**, (2022).

[13] Huang Y, He J, Zhu Z (2023). Rapid assessment of seismic risk for railway bridges based on machine learning. Int. J. Struct. Stab. Dy..

[14] Sresakoolchai J, Hamarat M, Kaewunruen S (2023). Automated machine learning recognition to diagnose flood resilience of railway switches and crossings. Sci. Rep. UK.

[15] Zhang S, Sheng D, Zhao G, Niu F, He Z (2016). Analysis of frost heave mechanisms in a high-speed railway embankment. Can. Geotech. J..

[16] Wan Z, Bian X, Chen Y (2022). Mud pumping in high-speed railway: in-situ soil core test and full-scale model testing. Railway Eng. Sci..

[17] Wang J, Li J, Li S (2023). figshare.

[18] Zhang G, Zheng D, Tian Y, Li S (2019). A dataset of distribution and diversity of ticks in China. Sci. Data..

[19] Zhang Q (2022). A dataset of distribution of antibiotic occurrence in solid environmental matrices in China. Sci. Data..

[20] Guo Y, Sun Q, Sun Y (2023). Dynamic evaluation of vehicle-slab track system under differential subgrade settlement in China's high-speed railway. Soil Dyn. Earthq. Eng..

[21] Wang J, Zhou Y, Wu T, Wu X (2019). Performance of cement asphalt mortar in ballastless slab track over high-speed railway under extreme climate conditions. Int. J. Geomech..

[22] Wang Z, Ling X, Wang L, Zhao Y, Cunha A (2021). Attenuation law of train-induced vibration response of subgrade in Beijing-Harbin railway. Shock Vib..

[23] Zhang Y, Sun B, Wen A, Cheng B (2019). Transverse thermal difference of high-speed railway roadbed in seasonally frozen regions. Proc. Inst. Civ. Eng. Gr..

[24] Liu H, Niu F, Niu Y, Xu J, Wang T (2016). Effect of structures and sunny–shady slopes on thermal characteristics of subgrade along the Harbin-Dalian Passenger Dedicated Line in Northeast China. Cold Reg. Sci. Technol..

[25] Li Z, Liu S, Feng Y, Liu K, Zhang C (2013). Numerical study on the effect of frost heave prevention with different canal lining structures in seasonally frozen ground regions. Cold Reg. Sci. Technol..

[26] Ouyang M, Hong L, Yu M-H, Fei Q (2010). STAMP-based analysis on the railway accident and accident spreading: Taking the China-Jiaoji railway accident for example. Saf. Sci..

[27] Kong L-W, Zeng Z-X, Bai W, Wang M (2017). Engineering geological properties of weathered swelling mudstones and their effects on the landslides occurrence in the Yanji section of the Jilin-Hunchun high-speed railway. B Eng. Geol. Environ..

[28] Wang S (2021). Application of Bayesian hyperparameter optimized random forest and XGBoost model for landslide susceptibility mapping. Front. Earth Sc-Switz..

[29] Habibi A, Delavar MR, Sadeghian MS, Nazari B, Pirasteh S (2023). A hybrid of ensemble machine learning models with RFE and Boruta wrapper-based algorithms for flash flood susceptibility assessment. Int. J. Appl. Earth Obs..

[30] Chen C (2023). Modeling landslide susceptibility in forest-covered areas in Lin’an, China, using logistical regression, a decision tree, and random forests. Remote Sens-Basel..

[31] Yichi Z, Hanping Z, Haoyue Q, Jinfan L (2023). Dynamic assessment of postdisaster road network vulnerability using crowdsourced traffic data. Transp. Res. D-Tr E..

[32] Kim J-C, Lee S, Jung H-S, Lee S (2017). Landslide susceptibility mapping using random forest and boosted tree models in Pyeong-Chang Korea. Geocarto Int..

[33] Wu X (2023). Analysis of geological hazard susceptibility of landslides in muli county based on random forest algorithm. Sustainability-Basel..

[34] Wang JF (2010). Geographical detectors-based health risk assessment and its application in the neural tube defects study of the Heshun region China. Int. J. Geogr. Inf. Sci..

[35] Hariharan S, Tirodkar S, Bhattacharya A (2016). Polarimetric SAR decomposition parameter subset selection and their optimal dynamic range evaluation for urban area classification using Random Forest. Int. J. Appl. Earth Obs..

[36] Kainthura, P. & Sharma, N. Hybrid machine learning approach for landslide prediction, Uttarakhand, India. *Sci Rep-UK*. **12**, (2022). 10.1038/s41598-022-22814-9 PMC9684430 36418362

[37] Zhang, T., Wang, D. & Lu, Y. Machine learning-enabled regional multi-hazards risk assessment considering social vulnerability. *Sci Rep-UK*. **13**, (2023). 10.1038/s41598-023-40159-9 PMC10435490 37591870

[38] Taalab K, Cheng T, Zhang Y (2018). Mapping landslide susceptibility and types using Random Forest. Big Earth Data..

[39] Yin, L., Zhu, J., Li, W., Wang, J. Vulnerability analysis of geographical railway network under geological hazard in China. ISPRS Int. J. Geo-Inf. **11**, (2022).

[40] Turner D, Lucieer A, Malenovský Z, King D, Robinson SA (2018). Assessment of antarctic moss health from multi-sensor UAS imagery with random forest modelling. Int. J. Appl. Earth Obs..

[41] Messina JP (2019). The current and future global distribution and population at risk of dengue. Nat. Microbiol..

[42] Wilschut LI (2013). Mapping the distribution of the main host for plague in a complex landscape in Kazakhstan: An object-based approach using SPOT-5 XS, Landsat 7 ETM+, SRTM and multiple Random Forests. Int. J. Appl. Earth Obs..

[43] Puissant A, Rougier S, Stumpf A (2014). Object-oriented mapping of urban trees using Random Forest classifiers. Int. J. Appl. Earth Obs..

[44] Zhang D (2022). A high-speed railway network dataset from train operation records and weather data. Sci. Data..

[45] Deng L (2022). Wireless monitoring of ballastless track slab deformation for high-speed railway. IEEE Trans. Instrum. Meas..

[46] Lai Y, Xu X, Dong Y, Li S (2013). Present situation and prospect of mechanical research on frozen soils in China. Cold Reg. Sci. Technol..

[47] Hong L, Ouyang M, Peeta S, He X, Yan Y (2015). Vulnerability assessment and mitigation for the Chinese railway system under floods. Reliab. Eng. Syst. Safe..

[48] Li G, Huang D, Gao W (2019). The analysis of ground subsidence along Jakarta-Bandung high-speed railway and main control measures. Railway Stand. Des..

[49] Chen D, Xu CA, Ni S (2016). Data mining on Chinese train accidents to derive associated rules. Proc. Inst. Mech. Eng..

[50] Wang F, Chen Y, Yan K (2023). A destructive mudstone landslide hit a high-speed railway on 15 September 2022 in Xining city, Qinghai province China. Landslides..

[51] Sun H, Di Z, Qin P, Zhang S, Lang Y (2024). Spatio-temporal variation and dynamic risk assessment of drought and flood disaster (DFD) in China. Int. J. Disast. Risk Red..

[52] Melnikov A, Zhang Z, Gagarin L (2023). Effects of extreme atmospheric precipitation on the stability of railways in the permafrost zone. Model Earth Syst. Env..

[53] Torvanger A, DyvikHenke C, Marginean I (2024). Improving climate risk preparedness - Railroads in Norway. Clim Serv..

[54] Sundell J, Haaf E, Tornborg J, Rosén L (2019). Comprehensive risk assessment of groundwater drawdown induced subsidence. Stoch. Env. Res. Risk A..

